# Striatal Neuron Excitability Is Regulated by Huntingtin in the Adult Brain

**DOI:** 10.1523/ENEURO.0269-25.2026

**Published:** 2026-06-09

**Authors:** Jessica C. Barron, Meghan L. Greenland, Samantha J. Carew, Fatemeh Ashrafganjoie, Emily P. Hurley, Christiana M. Kennedy, Firoozeh Nafar, Craig S. Moore, Matthew P. Parsons

**Affiliations:** Division of Biomedical Sciences, Faculty of Medicine, Memorial University, St. John’s, Newfoundland A1B 3V6, Canada

**Keywords:** electrophysiology, huntingtin, Huntington’s disease, neuroinflammation, neuronal excitation, striatum

## Abstract

Huntington's disease (HD) is a hereditary neurodegenerative disease that typically presents during midlife and is characterized by a combination of motor, cognitive, and psychiatric symptoms. HD is fatal and arises from a mutation in the huntingtin (*HTT*) gene, which results in decreased neuronal health followed by brain atrophy, with spiny projection neurons (SPNs) of the striatum being especially vulnerable to degeneration. HTT loss of function, caused by haploinsufficiency of the wild-type HTT gene (wt*HTT*), is an important feature of HD pathophysiology that has previously been understudied compared with mutant HTT gain-of-function mechanisms. wtHTT is essential for nervous system development and functions as a scaffolding protein to support many vital cellular functions including axonal transport, autophagy, and synaptic plasticity. Here, we examined the consequences of wtHTT deletion in the adult cortex and striatum by conditionally inactivating wtHTT in 2–4-month-old male and female *Htt*^fl/fl^ mice. wtHTT loss of function decreased intrinsic neuronal excitability within SPNs and produced a neuroinflammatory response in these mice, while tissue organization, spine morphology, and motor behavior remained unaffected. Results presented here provide additional evidence that wtHTT is vital for maintaining neuronal health in the adult brain and highlight some potential adverse consequences of nonselective HTT lowering for the treatment of HD.

## Significance Statement

Huntington's disease (HD) is a fatal brain disease caused by a mutation in the huntingtin (*HTT*) gene. Despite many ongoing clinical trials, there are currently no disease-modifying therapeutics available for HD patients. HTT-lowering therapeutics have shown promise as potential treatments for HD; however, many are nonselective for HTT and lower levels of mutant and wild-type HTT (wtHTT) proteins. Here, we sought to determine the consequences of wtHTT deletion in the adult cortex and striatum, regions particularly vulnerable to neurodegeneration in HD. wtHTT conditional knock-out decreased neuronal excitability and produced an inflammatory response, while tissue organization, spine dynamics, and motor phenotypes remained unaffected. Data presented here provide additional evidence that wtHTT is essential for neuronal health in the adult brain.

## Introduction

Huntington's disease (HD) is a monogenic neurodegenerative disease caused by an expansion of 35 or more CAG repeats in the huntingtin (*HTT*) gene, which leads to the production of a pathogenic mutant HTT protein (Bates et al., 2015). HD has an estimated prevalence of 13.7 per 100,000 in the general population (Fisher and Hayden, 2014), and individuals living with HD experience a triad of motor, cognitive, and behavioral symptoms, which typically present during middle age; however, symptom onset can vary from childhood to late adulthood (Walker, 2007). Despite many ongoing clinical trials, there is currently no cure for this fatal disease (Tabrizi et al., 2022). HD results in widespread atrophy throughout the brain, with spiny projection neurons (SPNs) of the striatum being particularly vulnerable (Vonsattel et al., 1985; Vonsattel and DiFiglia, 1998). In line with human pathology, numerous HD mouse models recapitulate SPN vulnerability (Hodgson et al., 1999; Slow, 2003; Heikkinen et al., 2012). Whole-cell recordings from mouse HD SPNs display alterations in excitatory activity early in the disease course (Li et al., 2004; Milnerwood et al., 2010, 2012). By DIV 21, SPNs cocultured with cortical neurons from the YAC128 HD mouse model have reduced dendritic arborization, fewer excitatory events, and fewer readily releasable pool synaptic vesicles (Buren et al., 2016). Ex vivo, many HD mouse models display similar alterations in SPN electrophysiological properties, such as fewer sEPSC and greater spontaneous IPSC events (Cummings et al., 2010). Comparable results are also seen in whole-cell recordings from striatal slices of Q175 knock-in HD mice (Indersmitten et al., 2015), highlighting these electrophysiological changes as a feature of HD brain pathology.

While mutation of the HTT protein has profound gain-of-function pathogenic effects on striatal neurons, loss of function of wild-type HTT (wtHTT) has also been shown to influence SPN health. For instance, striatal neurons do not produce the essential neurotrophin BDNF and therefore rely on its transport from the cortical projection neurons (Altar et al., 1997). wtHTT loss of function has been shown to downregulate the transcription of BDNF as well as disrupt its anterograde and retrograde axonal transport (Zuccato et al., 2001, 2003; Gauthier et al., 2004; Colin et al., 2008; Her and Goldstein, 2008). Nonpathogenic HTT is also known to have an antiapoptotic effect in HD culture and mouse models (Leavitt et al., 2001, 2006). Furthermore, previous research has shown that deletion of wtHTT in YAC128 mice worsened striatal atrophy, exacerbated motor deficits, and reduced survival rates (Van Raamsdonk et al., 2005a). Although overexpression of wtHTT reduced striatal atrophy, this did not translate to a complete rescue of motor deficits in YAC128 animals (Van Raamsdonk et al., 2006). We previously reviewed the extensive literature that supports the major role of wtHTT at the synapse (Barron et al., 2021). Notably, cortical deletion of wtHTT initially accelerated the rate of excitatory synapse formation in both the cortex and striatum, though this heightened excitatory drive was not maintained and reduced excitatory synapses were observed by 5 weeks of age (McKinstry et al., 2014). wtHTT is also a critical regulator of presynaptic neurotransmission in the striatum, as its deletion in striatal cultures disrupts synaptic vesicle endocytosis (McAdam et al., 2020). Other groups have investigated the embryonic deletion of wtHTT in distinct subpopulations of striatal neurons, such as in D1- and D2-expressing SPNs, and found it negatively impacted DARPP-32 levels and motor behavior (Mehler et al., 2019; Burrus et al., 2020). These studies provide compelling evidence for wtHTT as a necessary regulator of SPN survival in the HD brain.

Conditional deletion of wtHTT in the dorsal hippocampus of 2–4 month *Htt*^fl/fl^ mice results in profound morphological deficits and widespread reactive gliosis within 2 months (Barron et al., 2025a). wtHTT-depleted CA1 pyramidal neurons also had reduced intrinsic excitability, increased spontaneous EPSCs, and a loss of NMDAR-dependent long–term potentiation accompanied by impaired spatial memory (Barron et al., 2025a). These findings uncovered the novel role of wtHTT as an essential regulator of hippocampal synaptic plasticity and survival. As the striatum is a particularly susceptible brain area in HD and striatal SPNs are known to be negatively impacted when wtHTT is deleted embryonically, we hypothesized that wtHTT deletion in adulthood would negatively impact neuronal function. Here, AAV-Cre was injected into the striatum and overlying cortex to inactivate *HTT* in neurons of young adult male and female *Htt*^fl/fl^ mice. Two to three months following wtHTT conditional knock-out (cKO), we observed significant astrogliosis and decreased intrinsic excitability of SPNs, supporting the role of wtHTT in regulating neuronal physiology in the adult mammalian brain.

## Materials and Methods

### Animals

wtHTT cKO mice were initially provided by Dr. Scott Zeitlin (University of Virginia; Dragatsis et al., 2000). Breeding colonies were established and maintained at Memorial University of Newfoundland's Animal Care facility. Mice used in these experiments were generated from the C57BL/6J background strain and genetically edited to have their endogenous *wtHtt* alleles flanked with loxP sites to conditionally inactivate the mouse *Htt* gene in a homozygous manner by Cre expression (*Htt*^fl/fl^). Mice were bred as homozygous × homozygous. Equal numbers of male and female mice were used for all experiments. Mice were group housed in ventilated cage racks and kept on a 12 h light/dark cycle (lights on at 7:00 A.M.) with food and water available *ad libitum*. All animal procedures were performed in accordance with Memorial University of Newfoundland's animal care committee's regulations.

### Stereotaxic surgery

*Htt*^fl/fl^ mice aged 2–4 months were anesthetized using isoflurane (3% induction, 1.5–2% maintenance) and received a single subcutaneous injection of 2 mg/kg meloxicam in the abdomen and a single 0.1 ml/0.2% lidocaine injection below the scalp. Two small holes were drilled in the skull at the target brain coordinates using an Ideal Micro-Drill (Harvard Apparatus). A Model 7002 KH Neuros Hamilton Syringe and an infusion pump (Pump 11 Elite Nanomite, Harvard Apparatus) were used to inject 1 µl pENN.AAV.hSyn.HI.eGFP-Cre.WPRE.SV40 (Addgene, catalog #105540-AAV1) or 1 µl pAAV-hSyn-EGFP (Addgene, catalog #50465-AAV1) bilaterally into the dorsal striatum (injection rate, 2 nl/s). The following coordinates were used with respect to distance from bregma: 0.6 mm anterior, 1.8 mm medial/lateral, and 2.7 mm ventral to the brain surface. The syringe was left in place for at least 5 min before slow removal from the brain. Mice received a single 0.5 ml of 0.9% saline injection subcutaneously in the abdominal region, and the scalp incision was sutured. Mice recovered for 20–30 min postsurgery on a heating pad before being returned to their home cages. Surgeries were performed at regular intervals and alternated between male and female mice in order to prevent major differences in HTT knock-out duration between surgery and experimental endpoints. All animals underwent behavioral testing 1–2 months following stereotaxic surgery, and terminal experiments were performed at least 4 weeks after behavioral testing (2–3 months postsurgery).

### Western blotting

For Western blot experiments, the dorsal striatum of AAV-Cre-eGFP or AAV-eGFP-injected *Htt*^fl/fl^ mice were dissected and homogenized in 400 µl of lysis buffer containing halt protease and phosphatase inhibitor cocktails (Thermo Fisher Scientific, catalog #78440). The supernatant was collected, and the protein concentration was determined using bicinchoninic acid standards (Pierce Bicinchoninic Acid Protein Assay Kit, Thermo Fisher Scientific, catalog #23227). Protein samples were prepared by adding 4× lithium dodecyl sulfate sample buffer (Invitrogen, catalog #B0007) and heating at 70°C for 10 min. Samples were loaded onto a 4–12% Bis-Tris Plus gel (Invitrogen, catalog #NW04122BOX) for electrophoresis. Following electrophoresis, proteins were transferred onto a 0.45 µm nitrocellulose membrane (Santa Cruz Biotechnology, catalog #sc-201706) using wet transfer at 24 V for 16 h at 4°C. Transfer buffer contained 10% methanol. After transfer, the membrane was blocked with 5% nonfat milk in Tris-buffered saline with 0.05% Tween 20 for 1 h at room temperature to prevent nonspecific binding. Primary antibodies for anti-HTT (1:1,000; mouse, Sigma-Aldrich, catalog #MAB2166), anti-beta-actin (1:5,000, mouse, Sigma-Aldrich, catalog #A5316 clone AC74), and a goat anti-mouse IgG secondary antibody (1:5,000, Thermo Fisher Scientific, catalog #31430) were used. Blots were developed using a chemiluminescent horseradish peroxidase substrate (Super Signal West Pico Plus, Thermo Fisher Scientific, catalog #34580). Band densities were quantified in FIJI (Schindelin et al., 2012), and HTT band densities were normalized to actin.

### Acute slice preparation

Mice were anesthetized using isoflurane before decapitation and whole-brain removal*.* Brains were immersed in ice-cold oxygenated (95% O_2_/5% CO_2_) NMDG-HEPES artificial CSF slicing solution consisting of (in mM) 92 NMDG, 2.5 KCl, 1.25 NaH_2_PO_4_, 30 NaHCO_3_, 20 HEPES, 25 glucose, 2 thiourea, 5 Na-ascorbate, 3 Na-pyruvate, 0.5 CaCl_2_·2H_2_O, and 10 MgSO_4_·7H_2_O. NMDG solution was adjusted with HCl to reach a pH of 7.3–7.4. Dorsal striatal acute slices from one hemisphere (the opposite hemisphere was used for Golgi staining described on the next page) were sectioned using a Precisionary Compresstome. Slices were then transferred into warmed (32–34°C) NMDG-HEPES artificial CSF and underwent a sodium spike-in protocol (Ting et al., 2018). After this, slices recovered for 30–45 min in HEPES holding solution consisting of (in mM) 92 NaCl, 2.5 KCl, 1.25 NaH_2_PO_4_, 30 NaHCO_3_, 20 HEPES, 25 glucose, 2 thiourea, 5 Na-ascorbate, 3 Na-pyruvate, 2 CaCl_2_·2H_2_O, and 2 MgSO_4_·7H_2_O. HEPES artificial CSF solution was adjusted with NaOH to reach a pH of 7.3–7.4. Artificial CSF used during electrophysiological recordings consisted of (in mM) 125 NaCl, 2.5 KCI, 25 NaHCO_3_, 1.25 NaH_2_PO_4_, 1 MgCl_2_, 2 CaCl_2_, 10 glucose, and osmolarity adjusted to ∼310 mOsm.

### Whole-cell patch–clamp electrophysiology

Whole-cell patch–clamp recordings of green fluorescent protein (GFP)-positive cells were performed using 3–7 MΩ patch pipettes filled with potassium-gluconate internal recording solution made up of (in mM) 123 potassium-gluconate, 2 MgCl_2_, 1 KCl, 0.2 EGTA, 10 HEPES, 5 Na_2_ATP, and 0.3 Na_2_GTP, with osmolarity adjusted to 285 mOsm and containing Alexa Fluor 594 Hydrazide (Thermo Fisher Scientific, catalog #A10442) to visualize successfully patched neurons. Only GFP-expressing neurons were patched, and only cells that had electrophysiological properties typical of SPNs (e.g., hyperpolarized resting membrane potentials, inward rectification) were used for analysis. Input–output curves were obtained in current clamp mode, injecting current from −250 pA to +400 pA in 50 pA increments. Next, cells were held in voltage clamp (Vhold = −70 mV), and 2 min of stable recording was obtained to analyze sEPSC frequency and amplitude. Whole-cell recordings were analyzed using ClampFit version 10.6. Detected sEPSCs with amplitudes <5 pA were removed from the data analysis. Time between AAV injection and experimental endpoint (HTT knock-out duration) for animals used for whole-cell patch–clamp experiments averaged 83 d (mean ± 4.38 SEM).

### Golgi staining

Golgi staining was performed using Bioenno Lifesciences *sliceGolgi Kit* (catalog #003760) materials and protocol. Mouse hemibrains, dissected during striatal acute slice preparation, were submerged in a fixative buffer containing formaldehyde and glutaraldehyde. Brains remained in fixative solution at 4°C overnight. The fixed brain tissue was then coronally sectioned using a Precisionary Compresstome into 100 µm slices. Free-floating slices were incubated in Golgi impregnation solution for 7 d in the dark at room temperature. Slices were washed 1 week later with 0.01 M PBS-T and incubated in the remaining *sliceGolgi Kit* solutions. Striatal slices were next mounted onto gelatin-coated slides, allowed to dry completely, then dehydrated in graded ethanols, cleared using xylene and coverslipped with Permount mounting medium (Thermo Fisher Scientific, catalog #SP15-500). Golgi-stained dendrites from striatal neurons were imaged using an Axio Observer Z1 (Zeiss) microscope with a 63×/1.4NA oil-immersion objective (Zeiss). Image analysis was conducted using FIJI, where spine lengths and head widths were manually traced, and their measurements were quantified. Spine morphology was categorized using the following parameters: length <1 µm, stubby spine; length >1 µm and width <0.5 µm, thin spine; and length >1 µm and width >0.5 µm, mushroom spine (Risher et al., 2014). Time between AAV injection and experimental endpoint (HTT knock-out duration) for animals used for Golgi staining was the same as above, 83 d (mean ± 4.38 SEM), as these experiments used the opposite hemisphere as what was used for whole-cell patching.

### Histological staining

Mice were perfused intracardially using 4% paraformaldehyde to achieve fixation, and whole brains were dissected. Mice were held under anesthesia during the fixation procedure by the use of isoflurane (3% induction, maintained at 5% during perfusion). Fixed brains remained in 4% paraformaldehyde for 1 week and then briefly in 10% normal buffered formalin. Brains were then dehydrated starting with 70% ethanol, followed by 80%, 95%, and absolute ethanol, followed by 100% xylene before paraffin embedding. The paraffin-embedded brain tissue was then processed into 8 µm coronal sections using an automated vacuum infiltration tissue processor (TissueTek V.I.P. 5, Sakura Finetek). For the H&E protocol, slides were dipped in two changes of xylene for 5 min each. They were then rehydrated, starting with absolute ethanol, followed by 95, 80, and 70% ethanol and then washed with tap water for 2 min. Following rehydration, slides were stained with Mayer's hematoxylin solution (Millipore Sigma, catalog #109249) for 10–15 min and rinsed with tap water for 2 min. Slides were placed in Scott's tap water substitute for 2 min and washed with tap water for 5 min. Next, slides were counterstained in eosin for 15 s to 2 min. Slides were then dehydrated with 95% ethanol twice for 30 s each, followed by 30 s in absolute ethanol. Slides were cleared in three changes of xylene, 1 min each, and mounted with xylene-based mounting medium and coverslipped. Nuclei were stained blue, and cytoplasmic compartments were stained various shades of pink, which were used to identify tissue components. For the cresyl violet staining protocol, sections were deparaffinized and rehydrated as described above. Slides were then stained with cresyl violet stain solution (1%; Abcam, catalog #ab246817) for 3–5 min and rinsed with distilled water. Slides were then dehydrated with absolute ethanol, cleared with xylene, mounted and coverslipped as above. Nissl substance was stained violet, which was used to identify neuronal cell bodies. Images of H&E and cresyl violet stained slides were taken using an EVOS M5000 microscope (Thermo Fisher Scientific) with a 20×/0.8NA objective (Olympus). Neuropathology based on these histology stains was qualitatively assessed by a blind reviewer with histopathology training. Time between AAV injection and experimental endpoint (HTT knock-out duration) for animals used for histological experiments averaged 46 d (mean ± 1.04 SEM).

### Immunohistochemistry

A IHC hydrophobic barrier was drawn around striatal slides using a DAKO Pen (Agilent Technologies, catalog #s200230-2), which were briefly washed in 1× PBS solution. Striatal slides were then submerged in a blocking solution consisting of 5% bovine serum albumin and 0.2% Triton X-100 in 1× PBS for 1 h at room temperature. After blocking, primary antibodies (chicken anti-GFP, 1:500, Abcam, catalog #ab13970; mouse anti-GFAP, 1:500, Millipore Sigma, catalog #MAB3402; rabbit anti-IBA-1, 1:500, Cedarlane, catalog #019-19741) were diluted in a 1% bovine serum albumin/0.2% Triton X-100 PBS solution and incubated overnight on at 4°C. On the second day, slides were washed with 1× PBS and secondary antibodies (Alexa Fluor 488, goat anti-chicken, 1:500, Invitrogen, catalog #A-11039; Alexa Fluor 594, goat anti-rabbit, 1:500, Invitrogen, catalog #A-11037; Alexa Fluor 647, goat anti-mouse, 1:500, Invitrogen, catalog #A11029) diluted in a 1% bovine serum albumin/0.2% Triton X-100 PBS solution and incubated in the dark for 2 h at room temperature. Slides were then washed with 1× PBS and coverslipped with DAPI Fluoroshield mounting medium to visualize nuclei (Abcam, catalog #ab104139). All images were taken using either a Axio Observer Z1 (Zeiss) microscope with a 20×/0.8NA objective (Zeiss) or a EVOS M5000 (Thermo Fisher Scientific) microscope with a 20×/0.8NA objective (Olympus). Consistent imaging settings were used within each experiment. For GFAP and IBA-1 analysis, percent positive area values were quantified by applying a constant threshold value to all images. Percent positive area and mean gray (intensity) values were calculated using FIJI. Time between AAV injection and experimental endpoint (HTT knock-out duration) for animals used for IHC experiments averaged 102 d (mean ± 2.52 SEM).

### Behavior

#### Open field test (OFT)

All testing was done between 8:00 A.M. and 5 P.M. In all behavioral tests described, testing times were counterbalanced among groups. Time between AAV injection and behavioral testing (HTT cKO duration) for mice averaged 46 d (mean ± 1.23 SEM). All equipment was washed with 70% ethanol following each test. For OFT, mice were videotaped during a 10 min exploration of a brightly lit open field within a 50 × 50 cm white Plexiglass box. Distance traveled and mean velocity were scored using the ANYMaze software. Animals with <2 cm/s mean velocity were excluded from analysis for failing to explore the arena. Trials in which ANYMaze failed to track the animal >10% of the time were excluded from analysis.

#### Accelerated rotarod

Mice learned to run when placed on a rotating rod (rotarod) that accelerated from 4 to 40 rotations per minute for 300 s. When they fell, the experimenter stopped the rotarod manually. For some trials, the mice rotated around the rotarod instead of falling, which was considered equivalent to a fall. Mice performed three trials/day for 4 d with a 1.5–2 h intertrial interval. Latency to fall, number of rotations (around the rod), and number of turn arounds (where the mouse faces backward) were recorded.

### Statistics

All statistical tests were performed using version 10.2.2 GraphPad Prism. *N* values, specific statistical tests used, *p* values, *F* values, and degrees of freedom are described in Table 1. Outliers were identified using the ROUT method (Q = 1%). *P* values <0.05 were considered significant.

**Table 1.** Statistical tests used and accompanying values

Figure number	Experiment	Animal (*N*) and cell/image/slice number (*n*)	Statistics performed	*p* value
1*B*	HTT Western blot	*N* = 4, hemisphere *n* = 6–7	Unpaired parametric *t* test	CTL versus cKO: *p* = 0.0061
2*C*	Cell density (H&E)	*N* = 4, hemisphere *n* = 8	Unpaired parametric *t* test	CTL versus cKO: *p* = 0.104
2*C*	Cell density (Cresyl violet)	*N* = 4, hemisphere *n* = 8	Unpaired parametric *t* test	CTL versus cKO: *p* = 0.155
2*E*	Spine density	*N* = 4, dendrite *n* = 26	Unpaired parametric *t* test	CTL versus cKO: *p* = 0.4583
2*F*	Number of thin spines	*N* = 4, dendrite *n* = 26	Mann–Whitney test	CTL versus cKO: *p* = 0.1859
2*G*	Number of stubby spines	*N* = 4, dendrite *n* = 26	Mann–Whitney test	CTL versus cKO: *p* = 0.6129
2*H*	Number of mushroom spines	*N* = 4, dendrite *n* = 26	Mann–Whitney test	CTL versus cKO: *p* = 0.3819
2*J*	DAPI nuclear density	*N* = 4, image *n* = 15–16	Unpaired parametric *t* test	CTL versus cKO: *p* = 0.0865
3*B*	GFAP intensity	*N* = 4, image *n* = 16	Mann–Whitney test	CTL versus cKO: *p* = 0.0085
3*C*	GFAP % area	*N* = 4, image *n* = 12–14	Outliers excluded, Mann–Whitney test (cleaned data)	CTL versus cKO: *p* = 0.0750
3*D*	IBA-1 intensity	*N* = 4, image *n* = 16	Unpaired parametric *t* test	CTL versus cKO: *p* = 0.6575
3*E*	IBA-1% area	*N* = 4, image *n* = 13–15	Outliers excluded, Mann–Whitney test (cleaned data)	CTL versus cKO: *p* = 0.2352
4*B*	IV plot	*N* = 4–6, cell *n* = 13–22	Two-way ANOVA Repeated measures (mixed effects)	Genotype (CTL versus cKO): *p* = 0.0003
4*C*	Rheobase	*N* = 4–6, cell *n* = 13–22	Mann–Whitney test	CTL versus cKO: *p* = 0.0001
4*D*	Action potential threshold	*N* = 4–6, cell *n* = 13–22	Unpaired parametric *t* test	CTL versus cKO: *p* = 0.0002
4*E*	Resting membrane potential	*N* = 4–6, cell *n* = 13–22	Mann–Whitney test	CTL versus cKO: *p* = 0.0192
4*F*	Membrane resistance	*N* = 4–6, cell *n* = 13–22	Mann–Whitney test	CTL versus cKO: *p* = 0.0056
4*G*	Membrane capacitance	*N* = 4–6, cell *n* = 13–19	Outliers excluded, unpaired parametric *t* test (cleaned data)	CTL versus cKO: *p* = 0.5264
4*I*	sEPSC frequency	*N* = 4–6, cell *n* = 12–19	Mann–Whitney test	CTL versus cKO: *p* = 0.1770
4*J*	sEPSC IEI	*N* = 4–6, cell *n* = 12–19	Mann–Whitney test	CTL versus cKO: *p* = 0.1770
4*K*	sEPSC amplitude	*N* = 4–6, cell *n* = 12–19	Mann–Whitney test	CTL versus cKO: *p* = 0.0585
5*B*	OFT average distance	*N* = 20	Mann–Whitney test	CTL versus cKO: *p* = 0.5117
5*C*	OFT average speed	*N* = 20	Mann–Whitney test	CTL versus cKO: *p* = 0.5244
5*D*	Accelerating rotarod—latency to fall	*N* = 20	Two-way ANOVA	Genotype (CTL versus cKO): *p* = 0.8883

## Results

### Experimental design and validation of huntingtin cKO in adulthood

**Figure 1.**
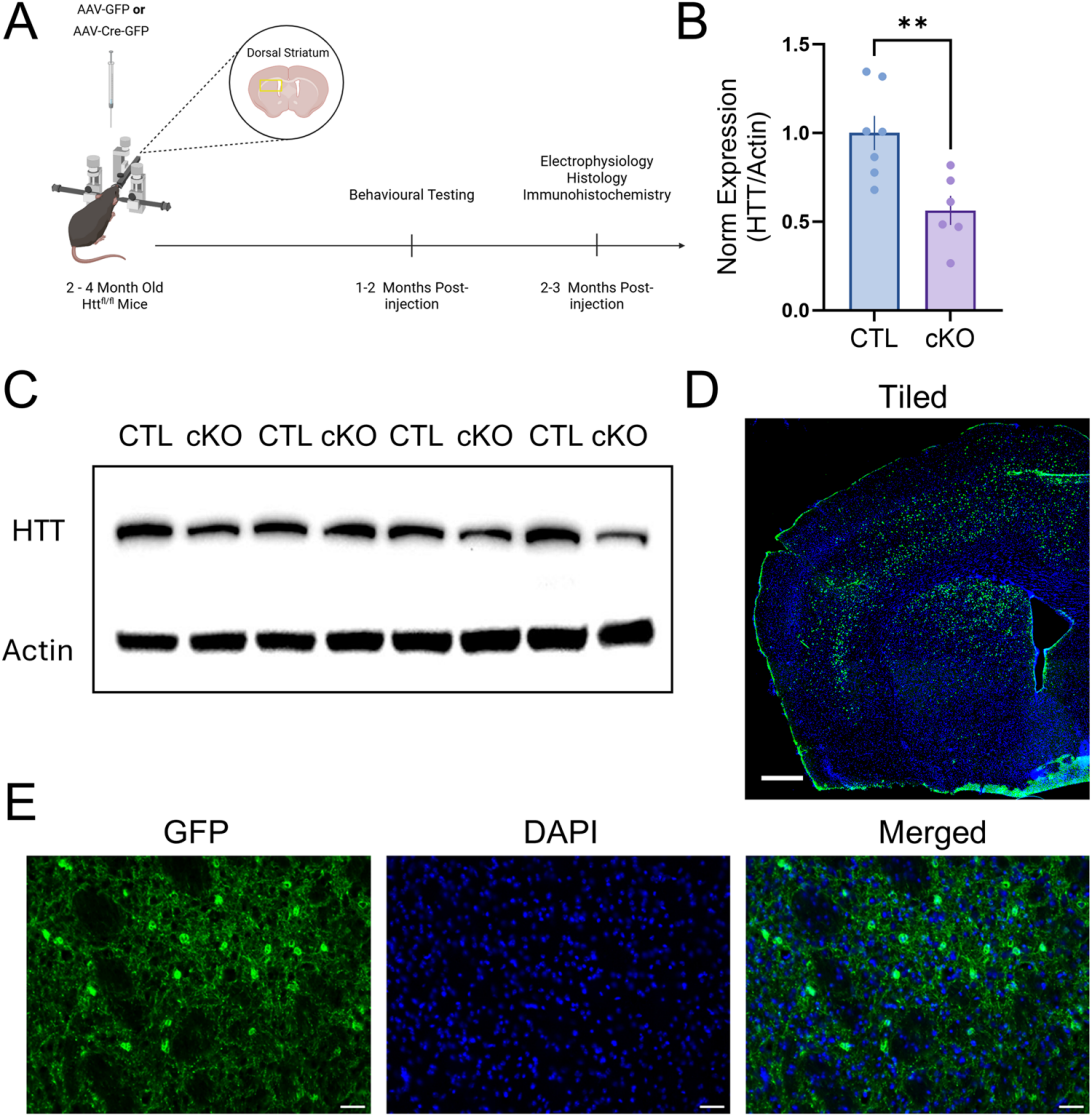
Experimental design and huntingtin knock-out validation. ***A***, Experimental methods and timeline. ***B***, Normalized HTT protein levels relative to actin control values. *N* = 4 animals. Data points represent individual hemispheres, with total protein samples obtained from the dorsal striatum. Representative Western blot shown in ***C***. ***D***, Tiled image demonstrating AAV-GFP-Cre expression (green) and DAPI-stained nuclei (blue) throughout the striatum and overlying cortex. Cortical GFP expression is also evident, demonstrating clear viral spread along the stereotaxic needle tract. Scale bar, 500 µm. ***E***, Representative images showing AAV-eGFP expression and DAPI-stained nuclei in the striatum. Scale bar, 25 µm. Unpaired parametric *t* test was used to determine statistical difference between groups. Data are represented as mean ± SEM. **p < 0.01.

To investigate the consequences of wtHTT knock-out in adulthood, synapsin-promoted AAV-eGFP-Cre or AAV-eGFP was injected into the dorsal striatum of 2–4-month-old male and female *Htt*^fl/fl^ mice. Viral targeting was confirmed by GFP expression. All behavioral testing took place 1–2 months postinjection, and brains were assessed using electrophysiological, histological, and immunohistochemical methods at 2–3 months postinjection (Fig. 1*A*). Successful reduction of the wtHTT protein was validated by Western blot analysis (Fig. 1*B*,*C*), with Cre-injected animals showing an average 43.6% reduction in HTT levels in total striatal lysates 1–2 months postsurgery (*p* = 0.0061). Despite injections being targeted to the dorsal striatum, we also observed widespread GFP-positive cells in the overlying cortex (Fig. 1*D*,*E*), likely resulting from viral spread along the stereotaxic needle tract during AAV delivery to the striatum. As a result, our wtHTT manipulation affects both the striatum and overlying cortex and is not exclusively striatal. A complete depletion of HTT was not expected in these total protein samples, as the tissue samples used included many non-neuronal cells that would still express normal levels of HTT.

### Huntingtin knock-out does not impact tissue organization in the mouse striatum but leads to astrogliosis

**Figure 2.**
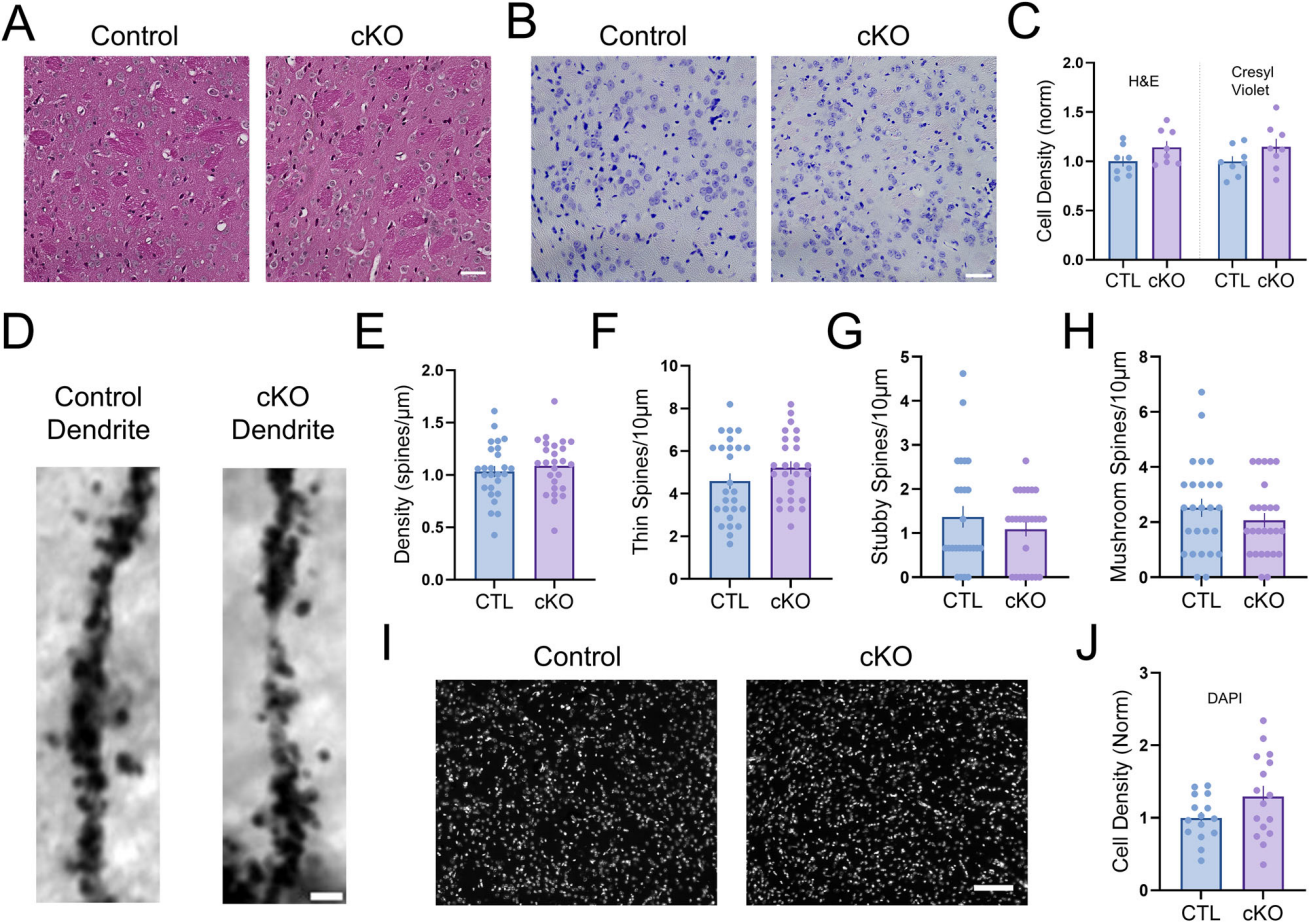
Neurohistolology of the mouse dorsal striatum following huntingtin cKO. ***A***, Representative control and wtHTT cKO slices histologically stained with hematoxylin and eosin (H&E) Scale bar, 30 µm. ***B***, Representative control and wtHTT cKO slices histologically stained with cresyl violet. Scale bar, 30 µm. ***C***, Quantification of cell density from H&E and cresyl violet staining. ***D***, Representative images of Golgi-stained dendrites from control and wtHTT cKO striatal slices. Scale bar, 2 µm. ***E***, Average spine density. ***F***, Average number of thin spines. ***G***, Average number of stubby spines. ***H***, Average number of mushroom spines. *N* = 4 animals. Data points represent average spine values per dendrite measured in 10 µm dendritic segments. ***I***, Representative images of control and wtHTT cKO slices stained with DAPI. ***J***, Analysis of nuclear density. Scale bar, 50 µm. *N* = 4 animals, each with bilateral AAV injections. Data points represent average nuclear density per image (2 images per hemisphere, sampled from the medial and lateral dorsal striatum). Unpaired parametric *t* test or Mann–Whitney test were used to determine statistical difference between groups, depending on data distribution. Data are represented as mean ± SEM.

We first wanted to determine how deletion of the wtHTT protein in adulthood would affect the general morphology of the striatum, as we previously demonstrated that wtHTT deletion in the adult mouse hippocampus caused a thinning of multiple CA1 layers (Barron et al., 2025a). Here, control and cKO H&E-stained slides were qualitatively assessed for changes in tissue organization and cell density, as well as for signs of inflammation or neurodegeneration. Morphology assessments described in this section focused on the upper half of the medial and lateral dorsal striatum, where viral injections were targeted. No obvious signs of neuropathology could be detected with either H&E staining (Fig. 2*A*) or cresyl violet staining (Fig. 2*B*), and no difference in cell density was observed in these histology sections (Fig. 2*C*; H&E *p* = 0.104; cresyl violet *p* = 0.155). Similarly, wtHTT cKO had no significant effect on nuclear density in the dorsal striatum (Fig. 2*I*,*J*; *p* = 0.0865). Spine density and morphology were then assessed by Golgi methods (Fig. 2*D*–*H*). No significant difference was seen in the spine density or morphology of striatal neurons (spine density *p* = 0.458; thin spines *p* = 0.186; stubby spines *p* = 0.613; mushroom spines *p* = 0.382). Dendritic complexity could not be accurately determined from our Golgi staining, due to a high level of dendritic overlap between neighboring neurons. Overall, 2–3 months wtHTT reduction did not impact tissue morphology or spine dynamics in the adult mouse brain.

**Figure 3.**
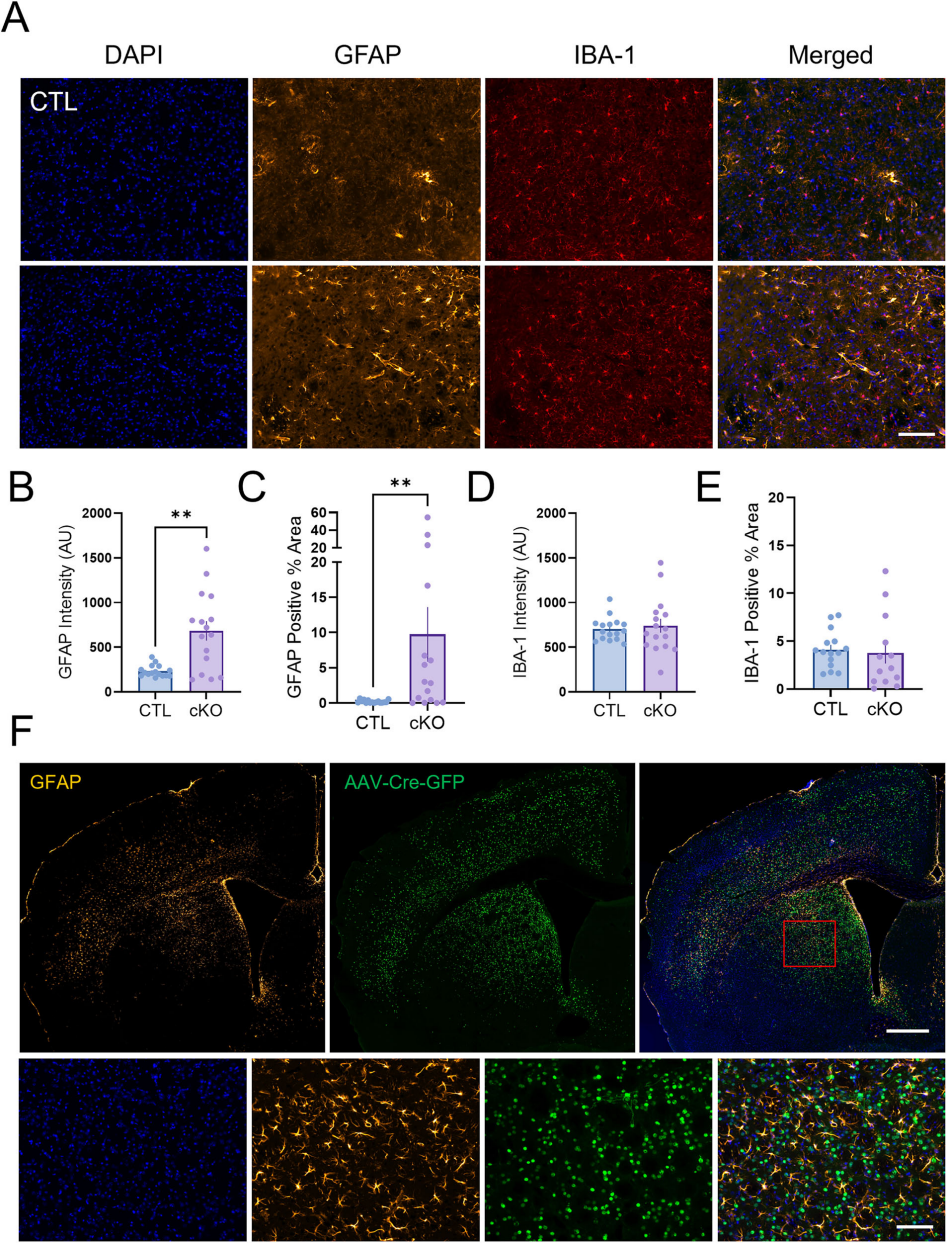
Huntingtin knock-out results in astrocyte gliosis in the dorsal striatum. ***A***, Representative images of control and wtHTT cKO slices immunohistochemically stained for glia analysis. Scale bar, 100 µm. ***B***, GFAP staining intensity. ***C***, The percentage of FOV with GFAP-positive signal. ***D***, IBA-1 staining intensity. ***E***, The percentage of FOV with IBA-1–positive signal. ***F,*** Representative tiled image showing GFAP, AAV-GFP-Cre, both channels merged with DAPI. Scale bar, 500 µm. Red square indicated the area enlarged in the lower panels. Scale bar, 100 µm. *N* = 4 animals. Data points represent average intensity or percentage area per image (2 images per hemisphere, sampled from the medial and lateral dorsal striatum). Unpaired parametric *t* test or Mann–Whitney test were used to determine statistical difference between groups, depending on data distribution. Data are represented as mean ± SEM. **p < 0.01.

The human HD striatum is marked by significant gliosis, which correlates with disease progression (Vonsattel et al., 1985). Here, wtHTT cKO increased GFAP intensity (Fig. 3*A,B*, *p* = 0.0085) and the percentage GFAP-positive area (Fig. 3*C*, *p* = 0.0067) in the mouse dorsal striatum. In contrast, IBA-1 immunostaining showed no significant difference in overall intensity or percentage positive area (Fig. 3*D*, *p* = 0.658; Fig. 3*E*, *p* = 0.235). The enhanced GFAP signal tended to spatially overlap with the distribution of AAV-Cre-GFP expression, suggesting that the astrocytic response was largely restricted to regions of wtHTT knock-out (Fig. 3*F*). These results suggest that wtHTT loss of function may induce a reactive astrocyte phenotype, mirroring a key pathological hallmark of HD.

### Deletion of wild-type huntingtin decreases neuronal excitability in the mouse striatum

**Figure 4.**
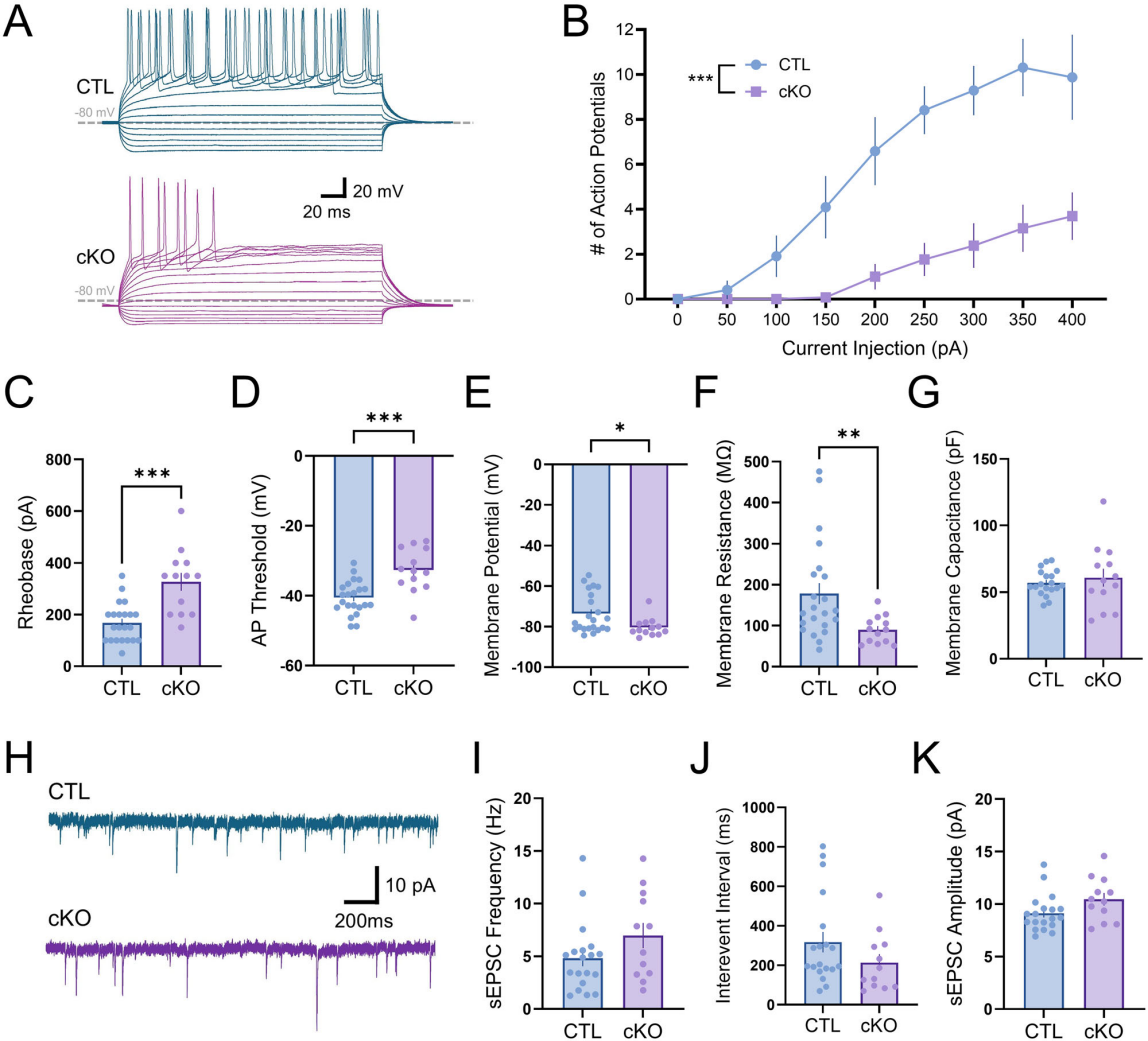
Deletion of huntingtin reduces intrinsic excitability in the mouse striatum. ***A***, Representative IV traces of control and wtHTT cKO neurons. ***B***, Current–voltage curve showing the average number of APs generated at each current sweep. ***C***, Average rheobase. ***D***, Average action potential (AP) threshold. ***E***, Average resting membrane potential. ***F***, Average membrane resistance. ***G***, Average membrane capacitance. ***H***, Representative sEPSC traces of control and wtHTT cKO neurons. ***I***, Average sEPSC frequency. ***J***, Average IEI. ***K***, Average sEPSC amplitude. *N* = 4 animals. Data points represent individual cells. Two-way RM ANOVA, unpaired parametric *t* test or Mann–Whitney test were used to determine statistical difference between groups, depending on dataset and distribution. Data are represented as mean ± SEM. *p < 0.05; **p < 0.01; ***p < 0.001.

Whole-cell patch clamping was used to investigate changes in cell excitability after wtHTT cKO in adult neurons. Here, wtHTT deletion resulted in extensive changes in intrinsic cell properties (Fig. 4*A*–*G*). Upon current injection, wtHTT cKO SPNs fired much fewer action potentials compared with their control GFP counterparts (Fig. 4*B*, *p* = 0.0003), which was accompanied by an increased average rheobase (Fig. 4*C*, *p* = 0.0001) and a depolarized action potential threshold (Fig. 4*D*, *p* = 0.0002). Other intrinsic neuronal properties were also impacted by wtHTT loss, including a hyperpolarized resting membrane potential (Fig. 4*E*, *p* = 0.0192) and a decrease in membrane resistance (Fig. 4*F*, *p* = 0.0056). Membrane capacitance remained unchanged, indicating cell size was not impacted by wtHTT depletion (Fig. 4*G*, *p* = 0.5264). Changes in sEPSCs in striatal neurons were also assessed after wtHTT loss (Fig. 4*H*–*K*). Data from these recordings show that the average frequency of spontaneous events and interevent interval (IEI) values were unchanged after wtHTT loss of function (Fig. 4*I*, *p* = 0.177; Fig. 4*J*, *p* = 0.177). However, a trending increase in sEPSC amplitude was observed (Fig. 4*K*, *p* = 0.0585). In sum, wtHTT deletion in adulthood altered numerous intrinsic properties of SPNs, significantly reducing their excitability.

### Deletion of wild-type huntingtin does not influence general motor activity or motor learning

**Figure 5.**
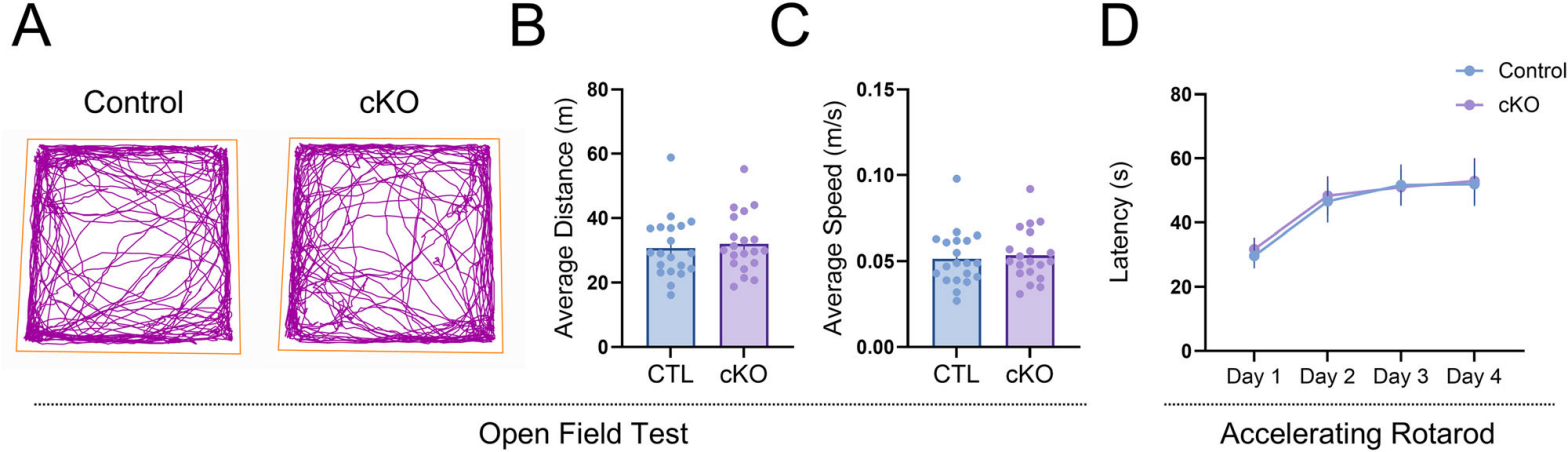
Huntingtin cKO does not influence general motor activity or motor learning. ***A***, Representative traces of ANYmaze video tracked OFT movements of control and wtHTT cKO mice. ***B***, Average total distance traveled during OFT. ***C***, Average speed during OFT. *N* = 20 animals. Data points represent individual animals. Unpaired parametric *t* test or Mann–Whitney test were used to determine statistical difference between groups, depending on data distribution. ***D***, Average latency to fall on rotarod during accelerated rotarod test. *N* = 20 animals. Data points represent average latency per genotype per trial. Two-way RM ANOVA was used to determine statistical difference between groups. Data are represented as mean ± SEM. Extended Data Figure 5-1 supports Figure 5.

Lastly, we assessed the impact of wtHTT deletion on motor function. Overall motor activity of control and cKO mice was quantified at 1–2 months postsurgery using an OFT paradigm where mice were tracked using the ANYmaze software (Fig. 5*A*). The background strain of these mice (C57BL/6J) have been previously reported to have sex-specific differences in their motor learning abilities, where 5-month-old males fell off the rotarod at significantly slower rotating speeds than female mice (Kovács and Pearce, 2013). Therefore, we also separated these datasets by sex, which can be viewed in the extended data (Extended Data Fig. 5-1). No sex differences in average distance or speed for the OFT were observed (Extended Data Fig. 5-1*A*,*B*). Average total distance and average speed during the 10 min testing trial were found to be comparable between control and wtHTT cKO mice, indicating that HTT depletion in striatal neurons did not impact general motor activity in these animals (Fig. 5*B*, *p* = 0.512; Fig. 5*C*, *p* = 0.5244). Next, mice were trained to run on an accelerating rotarod. This behavioral test is commonly used to quantify motor skill learning, and HD mice have significant impairments in rotarod motor learning (Van Raamsdonk et al., 2005b). For these experiments, mice were trained three times a day for 4 d, and latency to fall during each session was recorded. Both wtHTT cKO and control animals learned this behavioral test, with females performing statistically better overall compared with males, similar to what has been previously reported (Fig. 5-1*C*, *p* = 0.0062; Kovács and Pearce, 2013). However, wtHTT deletion had no effect on rotarod performance over the training sessions (Fig. 5*D*, *p* = 0.888). In all, these data show that motor behavior was comparable between control and cKO animals.

10.1523/ENEURO.0269-25.2026.f5-1 Figure 5-1 ***A,*** Average total distance travelled during OFT. ***B,*** Average speed during OFT. N = 10 animals. Data points represent individual animals. Data were assessed for sex differences by two-way RM ANOVA. D Average latency to fall on rotarod during accelerated rotarod test. N = 10 animals. Data points represent average latency per genotype/sex per trial. Two-way ANOVA was used to determine statistical difference between groups. Data are represented as mean ± SEM. Download Figure 5-1, TIF file.

## Discussion

Results presented here demonstrate that wtHTT deletion in cortical and striatal neurons of adult mice leads to reactive astrogliosis and decreased SPN excitability, while tissue morphology, spine morphology, and cell density in the striatum remained unchanged after 2–3 months of wtHTT loss. Additionally, no differences in general motor activity or motor learning were observed after 1–2 month conditional wtHTT deletion in these animals. Although previous HTT-lowering studies have reported that wtHTT deletion is well tolerated in the nonhuman primate brain (McBride et al., 2011; Grondin et al., 2012), significant inflammatory and electrophysiological changes were discerned in this cKO mouse model that may have been overlooked in previous studies evaluating the role of wtHTT in the adult brain.

### Huntingtin deletion in adulthood does not cause detectable neurodegeneration

Striatal neurodegeneration is a pathological hallmark of HD. The caudate and putamen structures that together make up the striatum of the basal ganglia undergo extensive volume loss, mainly due to the death of GABAergic SPNs, which comprise roughly 95% of neurons in this brain region (Vonsattel et al., 1985; Vonsattel and DiFiglia, 1998). Although HTT is ubiquitously expressed throughout the brain, striatal neurons exhibit modest expression levels of HTT compared with other brain areas like the cortex and the CA2/CA3 hippocampus (Reiner et al., 2003). Striatal SPNs are especially vulnerable to the expression of mutant HTT, and many hypotheses regarding this distinct susceptibility have been reviewed previously (Han et al., 2010). One prominent theory is that SPNs are particularly impacted by transcriptional dysregulation in HD and undergo significant loss of cell type identity (Malaiya et al., 2021; Obenauer et al., 2022; Matsushima et al., 2023). Based on their unique susceptibility to cell death in HD, it was anticipated that wtHTT deletion may lead to atrophy that would be detectable at the tissue level. Surprisingly, wtHTT knockdown in this model showed no differences in qualitative analysis of tissue morphology and quantitative analysis of nuclear density compared with controls. Similar studies have also reported that striatal morphology is unchanged after tamoxifen-induced wtHTT lowering in wild-type mice (Dietrich et al., 2017). Regio et al. recently found that when wtHTT was deleted by Cas9 gene editing in adult wild-type FVB mice, transcriptional profiles of these animals were largely comparable to controls (Regio et al., 2023). Striatal brain morphology was also normal in these animals, similar to what was observed here (Regio et al., 2023). Thus, transcriptional dysregulation and neurodegeneration in the HD striatum may therefore be driven primarily by gain-of-function mechanisms rather than wtHTT loss of function. In contrast, we have previously shown that wtHTT reduction in primary hippocampal neurons results in a loss of transcriptional repressive marks and altered chromatin morphology, suggestive of cell type-specific effects of wtHTT loss (Barron et al., 2025b).

### Reactive astrogliosis after 2–3 months of huntingtin knock-out

Starting early in the HD disease course, there is an increase in intensity levels of GFAP and IBA-1, astrocyte and microglia markers, respectively, and these glial cell types exhibit altered morphology (Dowie et al., 2014). In line with this, HD patients and mouse models also display elevated levels of inflammatory cytokines and chemokines (Björkqvist et al., 2008; Wild et al., 2011). wtHTT loss of function has been previously implicated in HD neuroinflammation. For instance, wtHTT-lowered human–derived macrophages are functionally impaired and are more vulnerable to stress (O’Regan et al., 2020). Additionally, 9 months of global wtHTT deletion in adulthood results in extensive astrogliosis (Dietrich et al., 2017). Here, 2–3 month wtHTT cKO was sufficient to elicit widespread astrocyte reactivity in the mouse striatum. However, although we previously found that wtHTT deletion in the hippocampus increased IBA-1 presence (Barron et al., 2025a), no change in IBA-1 staining intensity was detected in the current study. It is possible that the onset of microgliosis was simply missed given that microglia are one of the first immune cell types to respond to injury in the brain, with astrocytes becoming active much later (Fawcett and Asher, 1999). Astrocytes form close associations with neurons to create tripartite synapses and are important regulators of synaptic neurotransmission (Araque et al., 1999). It was recently proposed that astrocytes may also regulate the intrinsic excitability of neurons, as activation of hippocampal astrocytes led to a decrease in neuronal intrinsic excitation through adenosine signaling (Expósito et al., 2025). This could be a possible mechanism underlying the change in neuronal excitability seen in both HTT cKO mouse models.

### Huntingtin deletion in adulthood reduces striatal neuron excitability

The most striking finding of the present study was the impact of wtHTT deletion on striatal neuron excitability. Compared with controls, SPNs from cKO mice required significantly more current to elicit action potentials; an effect likely driven by the observed combination of a hyperpolarized membrane potential, a decreased membrane resistance, and a depolarized action potential threshold. While our AAV injection encompassed both cortical and striatal tissue (Extended Data Fig. 1-1*A*), the altered intrinsic properties observed here may reflect cell-autonomous effects of wtHTT loss in SPNs rather than secondary effects of wtHTT loss in striatal-projecting cortical neurons. Specifically, the decreased membrane resistance is suggestive of altered tonic currents in SPNs, such as an increase in potassium conductance, which would also account for the hyperpolarized membrane potentials. The depolarized action potential threshold suggests there may be altered expression, distribution, or biophysical properties of voltage-sensitive sodium channels within the recorded SPNs, rather than a network-mediated effect (Bean, 2007; Kole and Stuart, 2012). Furthermore, the lack of significant effect on sEPSC frequency suggests that there was no major change in excitatory drive onto striatal SPNs, much of which is of cortical origin (Hunnicutt et al., 2016). Thus, while we cannot entirely rule out the possibility that the observed changes in SPN excitability are driven downstream of altered corticostriatal synaptic activity, our data suggest that wtHTT expression in adulthood is required for SPNs to maintain their characteristic electrophysiological properties. Interestingly, our results differ considerably compared with published literature in various HD models, where decreased potassium channel expression and increasing membrane resistance typically lead to SPN hyperexcitability (Klapstein et al., 2001; Ariano et al., 2005a,b; Raymond et al., 2011; Cao et al., 2015). Thus, wtHTT loss of function and mHTT gain of function may exert opposing effects on SPN physiology.

A limitation of this work is that we did not distinguish between direct and indirect pathway subtypes; thus, our analysis reflects a mixed population of SPNs. Interestingly, prior work has demonstrated pathway-specific consequences of wtHTT loss on striatal SPN output (Burrus et al., 2020). Specifically, wtHTT deletion from indirect pathway SPNs reduced IPSC frequency in the globus pallidus externus, whereas wtHTT deletion from direct pathway SPNs increased IPSC frequency in the substantia nigra pars reticulata, indicating divergent downstream effects depending on SPN subtype (Burrus et al., 2020). While we were unable to distinguish SPN subtypes in the present study, we did not observe a bimodal distribution of intrinsic properties from recorded cKO neurons, arguing against opposing effects of wtHTT deletion on SPN subtypes. However, it is possible that our recordings were biased toward a specific cell type, potentially masking any pathway-dependent differences. Future studies incorporating D1- or D2-specific reporter lines are required to definitively determine if the hyperexcitable profile observed here is pathway specific.

Despite clear evidence of reduced SPN excitability, we did not observe any changes in motor activity were in the present study. Previous studies investigating the effect of wtHTT loss in adulthood on motor behavior have yielded mixed results. Global deletion of wtHTT between 3 and 9 months in mice elicited progressive motor deficits, including decreased latency to fall from an accelerated rotarod (Dietrich et al., 2017). In contrast, wtHTT knock-out more specifically targeted to the adult striatum and projecting areas showed no behavioral deficits in a comprehensive motor assessment (Regio et al., 2023). Therefore, the previously identified effects on motor performance may be due to wtHTT lowering in another brain area downstream from the striatum, such as the cortex.

## Conclusion

Overall, wtHTT conditional deletion in adult striatal and cortical neurons decreased SPN excitability and produced an astrogliosis response as quantified by GFAP staining, while tissue organization, spine morphology, cell density, and motor phenotypes remained unaffected. Results presented here contribute additional evidence to the growing body of literature that demonstrate wtHTT loss of function negatively impacts cellular function in the adult brain and caution that substantial reduction of wtHTT levels through the use of nonselective HTT-lowering therapeutics may have adverse molecular consequences such as those identified here.

## Synthesis

Reviewing Editor: Arvind Kumar, KTH Royal Institute of Technology Decisions are customarily a result of the Reviewing Editor and the peer reviewers coming together and discussing their recommendations until a consensus is reached. When revisions are invited, a fact-based synthesis statement explaining their decision and outlining what is needed to prepare a revision will be listed below. The following reviewer(s) agreed to reveal their identity: Margaux Lebouc.

Synthesis

The two reviewers appreciate the work as it gives insights into how huntingtin may affect neuronal properties. However, they have raised some concerns e.g. about the variance in the age of animals, data presentation in the figures (e.g. the full extent of GFP-positive cells is not visible) and data analysis (i.e. pooling together D1 and D2 SPNs). They have made a number of suggestions to improve the results and presentation of the results. I would like to invite you to revise your manuscript according to the suggestions of the reviewers.

Reviewer #1

The study titled, 'Striatal neuron excitability is regulated by huntingtin in the adult brain', investigates the impact of conditional huntingtin knockout in the striatum of adult mice. The authors look at the impact of this manipulation from the cellular level working up to behavioural tests. Given the current huntingtin lowering therapeutic approaches, it is important to determine the effect of loss of the wild type protein on cellular function. The authors show that there is altered function, particularly on the electrical properties of the slices, suggesting that huntingtin plays a role in controlling neuronal (and circuit) excitability.

Overall the experiments appear well conducted although there should be a greater level of detail included in all of the figure legends. To aid reader understanding the legends should have more experimental detail as to what was conducted as well as a greater level of detail regarding the statistics and the information presented. Specifically, an explanation as to what the n numbers are that are presented - are these individual animals/slices/coverslips/sweeps etc? This is particularly important for transparency of the statistics used.

The paper details that the experiments were carried out on 2-4 month old animals and the time between surgeries and experiments 1-2 months. A slightly variable age range in starting point is fairly normal but 1-2 months between surgeries and experiments seems quite a wide time window given the oldest animals would have been without huntingtin for twice the amount of time as the youngest potentially. How does this contribute to variability in the data and has this been controlled for in the comparisons?

The ephys characterisation experiments suggest a decrease in excitability of SPNs, what happens downstream of the SPNs, does this impact the post-synaptic response of the neurons the SPNs project to?

Minor points:

• Start of the results section references Fig1A where the experimental paradigm is Fig 1B (and vice versa for the first data panel).

• Fig 1: experiment validation - although there is clearly a lot of GFP positive cells overlapping with DARPP32 positive cells, it is not clear how extensive this is. It would be helpful to see a more zoomed out section to clearly show that the area targeted is the only area with GFP positive cells.

• Fig 2: some form of quantification for A and B would be helpful.

• The graph panels in Figure 2 are presented as part of panel C but in other figures, each graph has its own letter - this should be consistent.

• The behavioural data is presented as male and female in the appendix but this is not really commented on for the other figures. Assuming that there is no difference? Although in the paper the authors do comment that female mice are able to learn the tasks quicker, there is not any comment about the fact that it looks like the female cKO animals are the best - probably not something that you would predict and so would be interesting to know what the thoughts on that were. Related, to my point above, was there any difference in the time frame of the htt cKO was present - presumably the the cKO females did not have the longest time between surgery and experiment?

• The first line of the discussion talks about reactive gliosis - perhaps clarify this as there was no change in IBA1, only the astrogliosis that was increased.

Reviewer #2

Overall, the study investigates the effects of conditional wtHTT deletion in the adult striatum. The authors examined behavior, striatal morphology, and SPN excitability, and observed a decrease in SPN intrinsic excitability as well as astrogliosis. Given this, I have several comments and suggestions that I believe are essential to strengthen the manuscript:

Figure 1

Please provide a low-magnification image to visualize the full extent of GFP-positive cells and the spread of transduction in the DMS and DLS.

Figure 2

The authors state that H&E- and cresyl violet-stained sections were qualitatively assessed with no signs of neuropathology. However, without quantitative analysis (e.g. neuronal density, soma size), this conclusion is difficult to support. It is also unclear why cell density was measured using DAPI rather than directly from H&E or cresyl violet sections. The authors should either provide the quantitative data along with the images or avoid presenting images without quantification.

Did the authors measure the whole striatal area? If so, this should be stated in one sentence in the Results.

Was dendritic arborization assessed in the Golgi staining? If so, please state it in one sentence in the Results.

The authors indicate that "it was anticipated that wtHTT deletion would lead to striatal SPNs atrophy that would be detectable at the tissue level". However, they assessed cell density using DAPI, which labels all nuclei. DAPI alone cannot distinguish neurons from glia or other cell types, which could explain the non-significant trend observed. To clarify these results, I strongly recommend using SPN-specific markers such as Ctip2.

Figure 3

Additional images would strengthen the results. A low-magnification overview of the striatum should be included to locate the zoomed regions, followed by high-magnification images (e.g., x63) of the labeling.

Is the observed astrocytic gliosis restricted to the dorsal STR, or does it spread across the whole striatum?

How do the authors reconcile their findings with Regio et al., 2019, who did not observe changes in GFAP or Iba1 labeling?

Figure 4

Since the virus used is pan-neuronal, it is essential to specify whether only SPNs were patched and whether interneurons were excluded. Please clarify in the Methods.

D1- and D2-SPNs have well-established differences in electrophysiological properties (Cepeda et al., 2008; Gertler et al., 2008), with D2-SPNs generally being more excitable. To attribute the observed excitability changes specifically to wtHTT deletion, the two populations must be analyzed separately.

I also recommend separating sEPSC analysis by D1 vs D2 SPNs.

On line 329, please change 'MSN' to 'SPN' to maintain consistent terminology throughout the manuscript.

The key novel finding of this study is that conditional deletion of wtHTT in the adult striatum leads to a decrease in SPN intrinsic excitability. However, to fully support this conclusion, it is crucial to distinguish between D1- and D2-SPNs, given their distinct electrophysiological properties. Without this distinction, the interpretation of the functional data remains incomplete. I strongly recommend that the authors address this issue to ensure the validity of the main conclusion.
