# Supplementary figures and images for: Striatal Neuron Excitability Is Regulated by Huntingtin in the Adult Brain
Source: eNeuro. 2026 Jun 9;13(6):ENEURO.0269-25.2026. doi: 10.1523/ENEURO.0269-25.2026 (PMC13249443; doi:10.1523/ENEURO.0269-25.2026)

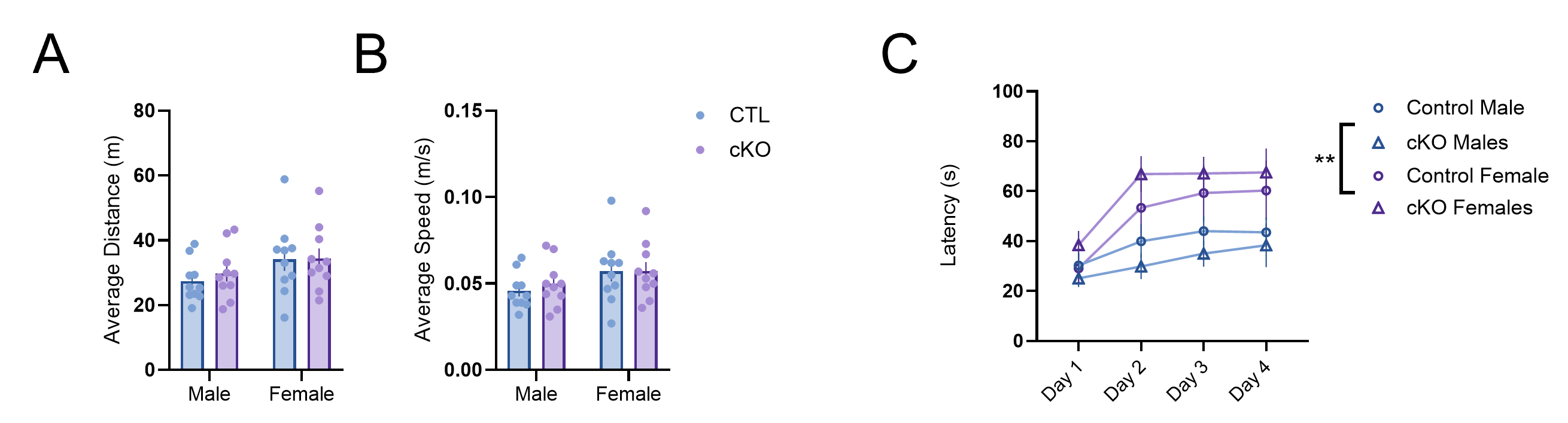

Supplement: Figure 5-1 — A, Average total distance travelled during OFT. B, Average speed during OFT. N = 10 animals. Data points represent individual animals. Data were assessed for sex differences by two-way RM ANOVA. D Average latency to fall on rotarod during accelerated rotarod test. N = 10 animals. Data points represent average latency per genotype/sex per trial. Two-way ANOVA was used to determine statistical difference between groups. Data are represented as mean ± SEM. Download Figure 5-1, TIF file. [file eneuro-13-ENEURO.0269-25.2026-s001.tif]
